# Novel Synthesis of Hydrazide-Hydrazone Derivatives and Their Utilization in the Synthesis of Coumarin, Pyridine, Thiazole and Thiophene Derivatives with Antitumor Activity

**DOI:** 10.3390/molecules16010016

**Published:** 2010-12-23

**Authors:** Rafat M. Mohareb, Daisy H. Fleita, Ola K. Sakka

**Affiliations:** 1 Department of Organic Chemistry, Faculty of Pharmacy, October University for Modern Sciences and Arts, October City, A.R., Egypt; 2 Department of Chemistry, Faculty of Science, Cairo University, Giza, A.R., Egypt; 3 Department of Chemistry, Faculty of Science, American University in Cairo, 5th Settlement, A.R., Egypt

**Keywords:** hydrazide-hydrazone, 3-acetylpyridine, thiophene, coumarin, antitumor activity

## Abstract

The reaction of cyanoacetyl hydrazine (**1**) with 3-acetylpyridine (**2**) gave the hydrazide-hydrazone derivative **3**. The latter compound undergoes a series of heterocyclization reactions to give new heterocyclic compounds. The antitumor evaluation of the newly synthesized products against three cancer cell lines, namely breast adenocarcinoma (MCF-7), non-small cell lung cancer (NCI-H460) and CNS cancer (SF-268) was performed. Most of the synthesized compounds showed high inhibitory effects.

## 1. Introduction

Hydrazines and their derivatives constitute an important class of compounds that has found wide utility in organic synthesis [1,2]. While hydrazines have traditionally been employed as reagents for the derivatization and characterization of carbonyl compounds, in recent years the N-N linkage has been used as a key structural motif in various bioactive agents. In particular, an increasing number of N-N bond-containing heterocycles and peptidomimetics have made their way into commercial applications as pharmaceutical and agricultural agents [3,4]. Recently, hydrazide-hydrazones have gained great importance due to their diverse biological properties including antibacterial, antifungal, anticonvulsant, anti-inflammatory, antimalarial and antituberculosis activities [5,6,7,8,9,10,11,12,13,14,15,16,17]. With the aim of obtaining novel hydrazide-hydrazones with a wide spectrum of pharmaceutical applications, we report herein the synthesis of a series of hydrazide-hydrazones together with their use in a series of heterocyclic transformations and their evaluation as anti-tumor agents [18,19,20,21]. 

## 2. Results and Discussion

**Scheme 1:**
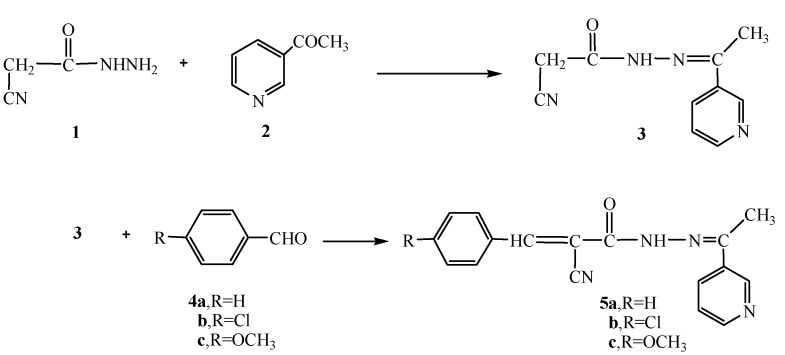
Synthesis of the hydrazide-hydrazones **3** and **5a-c**.

Recently, our research group became involved with a comprehensive program involving the synthesis of a series of hydrazide-hydrazone derivatives and their utilization in the synthesis of heterocyclic compounds with potential pharmaceutical and biological activities [22,23]. In continuation to this program, we report herein the reaction of cyanoacetylhydrazine (**1**) with3-acetylpyridine (**2**) in 1,4-dioxane to form the hydrazide-hydrazone derivative **3**. The structure of compound **3** was confirmed based on its analytical and spectral data. Thus, the ^1^H-NMR showed a singlet at δ 2.28 for the CH_3_ group, a singlet at δ 4.26 for the CH_2_ group, a multiplet at δ 7.43-8.99 for the pyridyl group and a singlet (D_2_O exchangeable) at δ 10.81 for the NH group. Moreover, the^13^C- NMR spectrum showed peaks at δ: 14.2 (CH_3_), 28.9 (CH_2_), 116.8 (CN), 122.1, 123.4, 133.7, 150.2, 151.0 (pyridine C), 168.1 (C=N), 169.8 (C=O). Further structure elucidation of compound **3 **was obtained through the study of its reactivity towards chemical reagents. Thus, the reaction of **3** with either benzaldehyde (**4a**), 4-chlorobenzaldehyde (**4b**) or 4-methoxybenzaldehyde (**4c**) gave the corresponding benzal derivatives **5a-c**, respectively (Scheme 1).

**Scheme 2:**
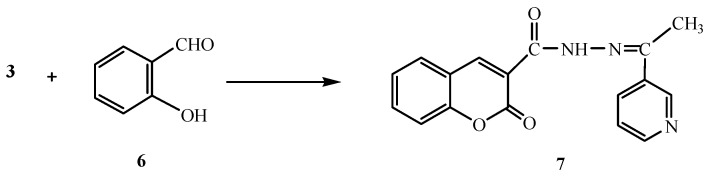
Synthesis of **7**.

On the other hand, the reaction of compound **3** with salicylaldehyde (**6**) gave the coumarin derivative **7** (Scheme 2). Analytical and spectral data of the product are in agreement with the proposed structure (see Experimental section).

**Scheme 3:**
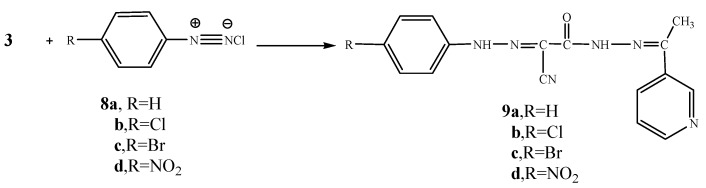
Synthesis of phenylhydrazo derivatives **9a-d.**

Next, we studied the reactivity of the active methylene group present in compound **3** towards diazonium salts. Thus, the reaction of **3** with either benzenediazonium chloride (**8a**), 4-chlorobenzene-diazonium chloride (**8b**), 4-bromobenzenediazonium chloride (**8c**) or 4-nitrobenzenediazonium chloride (**8d**), gave the hydrazone derivatives **9a-d**, respectively (Scheme 3). Analytical and spectral data of the latter reaction products are all consistent with the proposed structures.

**Scheme 4:**
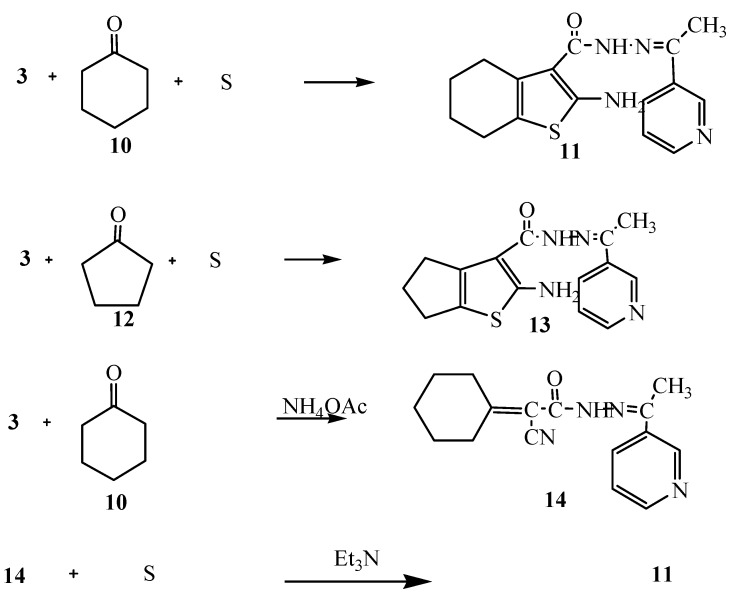
Synthesis of **11**, **13** and **14**.

Moreover, the reaction of compound **3** with cyclohexanone (**10**) and elemental sulfur in the presence of triethylamine was studied as an application of Gewald’s thiophene synthesis. The reaction led to the formation of 4,5,6,7-tetrahydrobenzo[*b*]thiophene derivative **11**. On the other hand, the reaction of compound **3** with cyclopentanone (**12**) and sulfur gave the cyclopentene[*b*]thiophene derivative **13**. The structures of compounds **11** and **13** were based on analytical and spectral data (see Experimental section). Further confirmation of structure **11** was obtained through its synthesis via another reaction route. Thus, the reaction of compound **3** with cyclohexanone in the presence of ammonium acetate in an oil bath at 140 °C gave the Knoevenagel condensation product **14**. The latter reacted with elemental sulfur in the presence of triethylamine to produce the same tetrahydrobenzo[*b*]thiophene derivative **11.** It is convenient to notice that the yield (70%) for formation of compound **11** using the latter procedure was higher than in the synthetic pathway described before (62%) (Scheme 4).

Next, we studied the reactivity of **3** towards phenyl isothiocyanate in basic dimethylformamide solution followed by heterocyclization with active methylene reagents like α-haloketones with the aim of synthesizing thiazole derivatives with potential antitumor activity. Thus, compound **3** reacted with phenylisothiocyanate (**15**) in DMF/KOH solution at room temperature to give the intermediate potassium sulphide salt **16**. Heterocyclization of **16** with α-haloketones like ethyl bromoacetate (**17**) gave the thiophene derivative **18**. The structure of **18** was confirmed based on analytical and spectral data. The ^1^H-NMR showed a singlet at δ 2.22 for the CH_3_ group, a singlet at δ 7.46 for the thiazole hydrogen, a multiplet at δ 7.54-8.95 for the pyridine H and C_6_H_5_ and two singlets at δ 8.80/10.13 for the NH and OH groups, respectively. Moreover, the ^13^C-NMR spectrum showed the presence ofpeaks at δ 14.3 (CH_3_), 93.0, 101.2 (C=C), 115.8 (CN), 119.2, 120.5, 121.8, 122.3, 124.0, 124.8, 127.6, 130.5, 133.2, 137.0, 150.7, 152.2 (C_6_H_5_, thiazole, pyridine C), 168.4 (C=N), 170.0 (C=O).

**Scheme 5:**
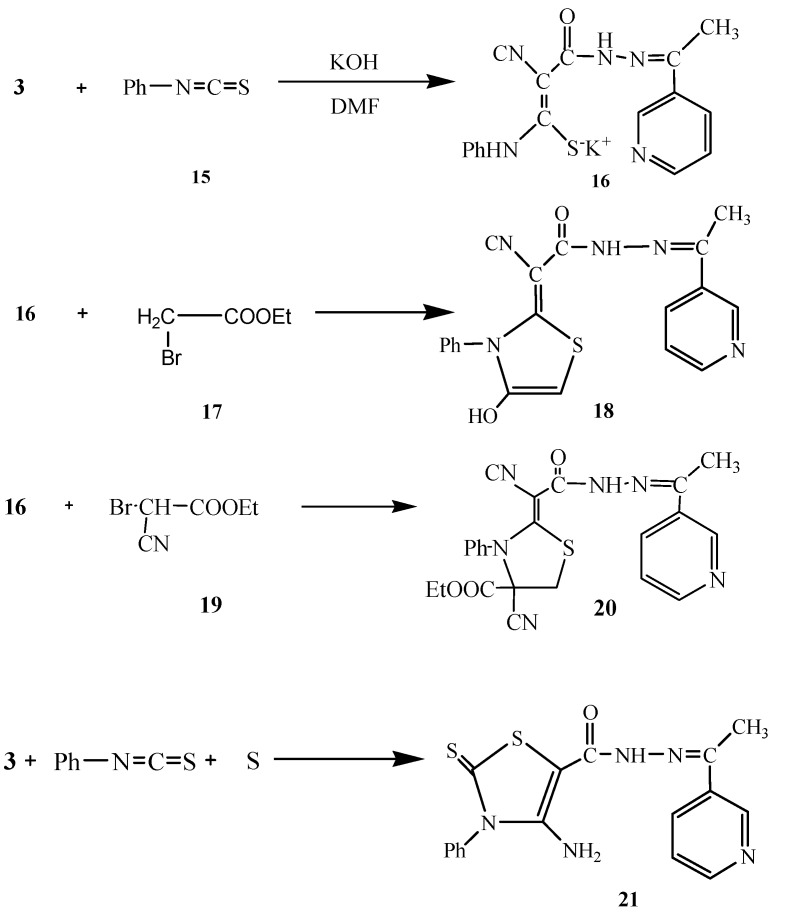
Synthesis of **18**, **20** and **21**

In a similar way, the reaction of **16** with ethyl bromocyanoacetate (**19**) gave the thiazole derivative **20**. Furthermore, compound **3** reacted with phenyl isothiocyanate and elemental sulfur in 1,4-dioxane containing triethylamine to give the thiazole derivative **21** (Scheme 5). The analytical and spectral data of **21** were in agreement with the proposed structure (see Experimental section).

**Scheme 6:**
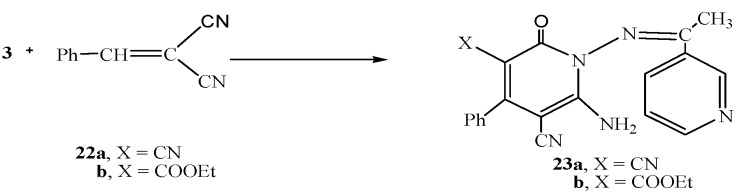
Synthesis of **23a,b**.

Next, we studied the reactivity of the hydrazide-hydrazone derivative **3** towards cinnamonitrile derivatives. Thus, the reaction of **3** with either 2-benzylidenemalononitrile (**22a**) or ethyl 2-cyano-3-phenylacrylate (**22b**) gave the pyridine derivatives **23a** and **23b**,respectively (Scheme 6).

### Effect on the Growth of Human Tumor Cell Lines

The effect of compounds **3-25** on the *in vitro* growth of three human tumor cell lines representing different tumor types, namely, breast adenocarcinoma (MCF-7), non-small cell lung cancer (NCI-H460) and CNS cancer (SF-268) was evaluated after a continuous 48 h exposure. The results are summarized in Table 1. All of the tested compounds were able to inhibit the growth of the tested human tumor cell lines in a dose-dependant manner (data not shown). The results indicated in Table 1 revealed that compound **3** showed the highest inhibitory effect against all the three tumor cell lines. In addition compound **5c **showed the best inhibitory effect against CNS cancer (SF-268), while compounds **7** and **23a** showed high inhibitory effects against non-small cell lung cancer (NCI-H460) and breast adenocarcinoma (MCF-7), respectively. Compounds **5b**, **9a**, **23b **showed the lowest inhibitory effects. The rest of the compounds showed a moderate growth inhibitory effect. Comparing compound **5b **with **5c**, it is obvious thatthe presence of the CH_3_O group in **5c **resulted in a higher inhibitory effect than **5b **with the Cl group. Comparing the pyridine derivatives **23a** (with the cyano group) and **23b **(with the carboxyethyl group), the first has a greater inhibitory effect than the second towards the three cell lines.

**Table 1:** Effect of the newly synthesized product on the growth of three human tumor cell.

GI50 (µM)			
Compound	MCF-7	NCI-H460	SF-268
**3**	0.1 ± 0.009	0.2 ± 0.001	0.6 ± 0.001
**5a**	20.0 ± 0.2	26.6 ± 1.4	38.4 ± 0.6
**5b**	60.6 ± 16.9	38.9 ± 10.8	28.8 ± 8.6
**5c**	2.0 ± 0.2	3.0 ± 1.6	0.07 ± 0.001
**7**	66.8 ± 12	10 ± 6.2	36.8 ± 3.0
**9a**	74.7 ± 17.5	48.2 ± 12.8	62.0 ± 9.01
**9b**	20 ± 0.4	20.3 ± 0.8	22.2 ± 0.8
**9c**	28.9 ± 0.9	40.6 ± 1.8	54.8 ± 0.8
**11**	30 ± 0.6	17.3 ± 1.4	22.3 ±1.5
**13**	36.0 ± 1.8	44.0 ± 0.8	20.5 ± 1.1
**14**	50.1 ± 0.7	23.2 ± 4.8	18.4 ± 1.8
**17**	22 ± 0.4	20.3 ± 0.8	30 ± 0.8
**19**	22.0 ± 0.2	24.1 ± 0.8	38.4 ± 0.6
**21**	35.4 ± 10.2	24.1 ± 0.8	18.9 ± 6.8
**23a**	11.9 ± 0.5	14.1 ± 0.6	20.3 ± 0.5
**23b**	70.9 ± 0.9	40.6 ± 1.8	60.8 ± 0.8

Results are given in concentrations that were able to cause 50 % of cell growth inhibition (GI_50_). after a continuous exposure of 48 h and show means ± SEM of three-independent experiments performed in duplicate. Doxorubicin was used as positive control, GI50: MCF-7 = 42.8 ± 8.2 nM; NCI-H460 = 94.0 ± 8.7 nM, and SF-280 = 94.0 ± 7.0 nM.

## 3. Experimental

### 3.1. General

Melting points were determined on an Electrothermal melting point apparatus (Electrothermal 9100) and are uncorrected. IR spectra were recorded for KBr discs on a Pye Unicam SP-1000 spectrophotometer. ^1^H-NMR and ^13^C-NMR spectra were measured on a Varian EM-390-200 MHz in CD_3_SOCD_3_ as solvent using TMS as internal standard, and chemical shifts are expressed as δ. Analytical data were obtained from the Microanalytical Data Unit at Cairo University, Giza, Egypt. Antitumor evaluation for the newly synthesized products were performed by a research group at the National Research Center and the National Cancer Institute at Cairo University.

*2-Cyano-N'-(1-(pyridine-3-yl)ethylidene)acetohydrazide* (**3**). To a solution of cyanoacetylhydrazine (**2**, 0.99 g, 0.01 mol) in 1,4-dioxane (20 mL), 3-acetylpyridine (1.21 g, 0.01 mol) was added. The reaction mixture was heated under reflux for 2 h then poured onto a beaker containing an ice/water mixture. The formed solid product was collected by filtration and dried to give white crystals (from ethanol). Yield: 1.50 g, 74%, m.p. 203-205 °C; IR (KBr) υ/cm^−1^: 3500-3400 (NH), 3066 (CH-aromatic), 2885 (CH_3_), 2200 (CN), 1680 (C=O); MS *m/z* (%) 202 [M^+^, 8%]; ^1^H-NMR δ: 2.28 (s, 3H, CH_3_), 4.26 (s, 2H, CH_2_), 7.43-8.99 (m, 4H, pyridine-H),10.81(s, 1H, NH); ^13^C-NMR δ: 14.2 (CH_3_), 28.9 (CH_2_), 116.8 (CN), 122.1, 123.4, 133.7, 150.2, 151.0 (pyridine C), 168.1 (C=N), 169.8 (C=O); Anal. Calcd. for C_10_H_10_N_4_O (202.21): C, 59.40; H, 4.98; N, 27.71%. Found: C, 59.72; H, 5.20; N, 28.01%. 

### 3.2. General Procedure for the Synthesis of *5a,* *5b* or *5c*

To a solution of **3** (2.02 g, 0.01 mol) in 1,4-dioxane (20 mL), either benzaldehyde (1.06 g, 0.01 mol), *p*-chlorobenzaldehyde(1.12 g, 0.01 mol) or *p*-methoxybenzaldehyde (1.08 g, 0.01 mol) was added. The reaction mixture was heated under reflux for 6 h then poured onto a beaker containing an ice/water mixture. The formed solid product was collected by filtration and dried.

*2-Cyano-3-phenyl-N'-(1-(pyridin-3-yl)ethylidene)acrylohydrazide* (**5a**). White crystals (from acetone). Yield: 1.32 g, 45.4%, m.p. 115-118 °C; IR (KBr) υ/cm^−1^: 3460-3380 (NH), 3050 (CH-aromatic), 2890 (CH_3_), 2220 (CN), 1683 (C=O), 1635 (C=N); MS *m/z* (%) 290 [M^+^, 39.7%];^1^H-NMR δ: 2.28 (s, 3H, CH_3_), 3.31 (s, 1H, CH), 7.30-7.91 (m, 9H, C_6_H_5_, pyridine-H), 8.71 (s, 1H, NH); ^13^C-NMR δ: 14.0 (CH_3_), 98.3 (C=CH), 148.0 (C=CH), 116.8 (CN), 124.3, 125.7, 15.9, 126.2, 128.0, 129.4, 136.5, 150.2, 151.6 (benzene, pyridine C), 165.3 (C=N), 170.0 (C=O); Anal. Calcd. for C_17_H_14_N_4_O (290.32): C, 70.33; H, 4.86; N, 19.30%. Found: C, 70.01; H, 4.92; N, 19.48%.

*3-(4-Chlorophenyl)-2-cyano-N'-(1-(pyridin-3-yl)-ethylidene)acrylo-hydrazide* (**5b**). Pale yellow crystals (from acetone). Yield: 1.95 g, 60.1%, m.p. 100 °C; IR (KBr) υ/cm^−1^: 3460-3380 (NH), 3050 (CH-aromatic), 2890 (CH_3_), 2200 (CN), 1683 (C=O), 1623 (C=N); MS *m/z* (%) 324 [M^+^, 5.17%];^1^H-NMR δ: 2.27 (s, 3H, CH_3_), 3.38 (s, 1H, CH), 7.17-7.94 (m, 8H, C_6_H_4_, pyridine-H), 10.00 (s, 1H, NH); ^13^C-NMR δ : 14.1 (CH_3_), 98.0 (C=CH), 147.8 (C=CH), 116.9 (CN), 122.8, 124.6, 15.9, 126.0, 127.1, 128.9, 136.5, 150.2, 151.6 (benzene, pyridine C), 165.3 (C=N), 170.0 (C=O); Anal. Calcd. for C_17_H_13_ClN_4_O (324.76): C, 62.87; H, 4.03; N, 17.25%. Found: C, 63.01; H, 4.26; N, 17.32%.

*2-Cyano-3-(4-methoxyphenyl)-N'-(1-(pyridin-3-yl)ethylidene)acrylo-hydrazide* (**5c**). Yellow crystals (from acetone). Yield: 2.40 g, 75%, m.p. 130-133 °C; IR (KBr) υ/cm^−1^: 3460-3370 (NH) , 3100 (CH-aromatic), 2890 (CH_3_), 2200 (CN), 1683 (C=O), 1640 (C=N); MS *m/z* (%) 320 [M^+^, 44.28%]; ^1^H-NMR δ: 2.49 (s, 3H, CH_3_), 3.31(s, 1H, CH), 3.82 (s, 1H, CH_3_), 7.03-7.82 (m, 8H, C_6_H_4_, pyridine-H), 8.62 (s, 1H, NH);^ 13^C-NMR δ: 14.4, 54.6 (2 CH_3_), 99.9. (C=CH), 148.3 (C=CH), 116.3 (CN), 122.3, 124.6, 125.3, 125.9, 127.8, 128.4, 135.3, 150.0, 151.9 (benzene, pyridine C), 165.6 (C=N), 168.9 (C=O); Anal. Calcd. for C_18_H_16_N_4_O_2_ (320.35): C, 67.49; H, 5.03; N, 17.49%. Found: C, 67.55; H, 4.89; N, 17.31%.

*2-Oxo-N'-(1-(pyridin-3-yl)ethylidene)-2H-chromene-3-carbohydrazide* (**7**). To a solution of **3 **(2.02 g, 0.01 mol) in 1,4-dioxane (20 mL), salicylaldehyde (1.22 g,0.01 mol) was added. The reaction mixture was heated under reflux for 3 hours then poured onto a beaker containing an ice/water mixture. The formed solid product was collected by filtration and dried to give white crystals (from ethanol). Yield: 2.10 g, 68%, m.p. 235-238 °C; IR (KBr) υ/cm^−1^: 3450-3360 (NH), 3200 (CH-aromatic), 2890 (CH_3_), 2200 (CN), 1670 (C=O), 1650 (C=N); MS *m/z* (%) 307 [M^+^, 2.5%]; ^1^H-NMR δ2.51(s, 3H, CH_3_), 7.30 (s, 1H, coumarin H-4), 7.48-9.33 (m, C_6_H_4_, pyridine-H), 13.69 (s, 1H, NH); ^13^C-NMR δ: 13.8 (CH_3_), 121.8, 122.6, 122.9, 124.5, 127.1, 128.0, 130.9, 131.6, 150.2, 152.3 (C_6_H_4_, pyridine C), 159.9 (coumarin C=O), 167.9 (C=N), 169.8 (C=O); Anal. Calcd. for C_17_H_13_N_3_O_3_ (307.30): C, 66.44; H, 4.26; N, 13.67%. Found: C, 66.38; H, 4.35; N, 13.92%.

### 3.3. General Procedure for the Synthesis of *9a, 9b, 9c* or *9d*

To a cold solution of **3 **(2.02 g, 0.01 mol) in ethanol (20 mL) containing sodium acetate (3.0 g) was added with continuous stirring either of the appropriate substituted benzenediazonium salt (0.01 mol) [prepared by adding sodium nitrite (1.6 g, 0.02 mol) in water (8 mL) to a cold solution of either of the appropriate substitute aniline **8a-d** in the appropriate amount of hydrochloric acid]. The reaction mixture was stirred for 2 h and the formed solid product, in each case, was collected by filtration.

*2-Cyano-2-(2-phenylhydrazinylidene)-N'-[1-(pyridin-4-yl)ethylidene]aceto-hydrazide* (**9a***).* Orange crystals (from ethanol). Yield: 1.92 g, 63%, m.p. 158-161 °C; IR (KBr) υ/cm^−1^: 3500-3400 (NH), 3050 (CH-aromatic), 2800 (CH_3_), 2200 (CN), 1696 (C=O); MS *m/z* (%) 306 [M^+^, 93.51%]; ^1^H-NMR δ2.49 (s, 3H, CH_3_), 7.11-8.22 (C_6_H_5_, pyridine H), 10.25, 12.02 (2s, 2H, 2NH); ^13^C-NMR δ: 14.1 (CH_3_), 116.0 (CN), 115.9, 116.8, 118.6, 122.3, 123.9, 129.0, 142.8, 150.2, 152.8 (C_6_H_5_, pyridine C), 163.2, 166.7 (2 C=N), 168.8 (C=O); Anal. Calcd. for C_16_H_14_N_6_O (306.32): C, 62.74; H, 4.61; N, 27.44%. Found: C, 62.90; H, 4.51; N, 27.52%.

*2-[2-(4-Chlorophenyl)hydrazinylidene]-2-cyano-N'-[1-(pyridin-4-yl)ethylidene] acetohydrazide* (**9b**). Orange crystals (from ethanol). Yield: 2.32 g, 68%, m.p. 174-177 °C; IR (KBr) υ/cm^−1^: 3600-3500 (NH), 3100 (CH-aromatic), 2890 (CH_3_), 2200 (CN), 1696 (C=O); MS *m/z* (%) 340 [M^+^, 6.17%]; ^1^H-NMR δ2.49 (s, 3H, CH_3_), 6.95-7.89 (C_6_H_4_-pyridine H), 11.737, 12.75 (2s, 2H, 2NH); ^13^C-NMR δ: 14.0 (CH_3_), 115.8 (CN), 116.0, 117.3, 118.9, 121.8, 124.5, 125.4, 143.5, 150.0, 151.8 (C_6_H_5_, pyridine C), 163.0, 166.9 (2 C=N), 168.9 (C=O); Anal. Calcd. for C_16_H_13_ClN_6_O (340.77): C, 56.39; H, 3.85; N, 24.66%. Found: C, 56.60; H, 4.01; N, 24.90%.

*2-[2-(4-Bromophenyl)hydrazinylidene]-2-cyano-N'-[1-(pyridin-4-yl)ethylidene] acetohydrazide* (**9c**). Orange crystals (from ethanol). Yield: 2.62 g, 68%, m.p. 183-186 °C; IR (KBr) υ/cm^−1^: 3550-3400 (NH), 3090 (CH-aromatic), 2890 (CH_3_), 2200 (CN), 1685(C=O); MS *m/z* (%) 387 [M^+^, 19 %]; ^1^H-NMR δ 2.49 (s, 3H, CH_3_), 6.93-7.84 (C_6_H_4_, pyridine H), 9.50, 11.75 (2s, 2H, 2NH); ^13^C-NMR δ: 14.2 (CH_3_), 116.6 (CN), 116.3, 118.0, 118.6, 122.8, 124.5, 128.0, 138.9, 150.2, 152.5 (C_6_H_5_, pyridine C), 163.0, 166.5 (2 C=N), 168.5 (C=O); Anal. Calcd. for C_16_H_13_BrN_6_O (384): C, 49.89; H, 3.40; N, 21.82%. Found: C, 48.93; H, 3.62; N, 22.02%.

*2-Cyano-2-[2-(4-nitrophenyl)hydrazinylidene]-N'-[1-(pyridin-4-yl)ethylidene] acetohydrazide* (**9d**). Orange crystals (from ethanol). Yield: 2.43 g, 69%, m.p. 162-165 °C; IR (KBr) υ/cm^−1^: 3570-3400 (NH), 3100 (CH-aromatic), 2890 (CH_3_), 2200 (CN), 1670 (C=O); MS *m/z* (%) 352 [M^+^, 20.2 %]; ^1^H-NMR δ 2.49 (s, 3H, CH_3_), 6.57-8.39 (C_6_H_4_, pyridine H), 10.48, 12.11 (2s, 2H, 2NH); ^13^C-NMR δ: 14.1 (CH_3_), 116.0 (CN), 116.8, 116.9, 118.2, 123.8, 126.3, 128.6, 136.1, 151.0, 152.7 (C_6_H_5_, pyridine C), 163.1, 166.4 (2 C=N), 168.9 (C=O); Anal. Calcd. for C_16_H_13_N_7_O_3_ (351.32): C, 54.70; H, 3.73; N, 27.91%. Found: C, 54.85; H, 3.90; N, 29.11%.

*2-Amino-4,5,6,7-tetrahydro-N'-(1-(pyridin-3-yl)ethylidene)benzo[b]thiophene-3-carbohydrazide* (**11**). Method A: To a solution of **3 **(2.02 g, 0.01 mol) in ethanol (40 mL) containing triethylamine (1 mL) and elemental sulfur (0.32 g, 0.01mol), cyclohexanone **10** (0.98 g, 0.01 mol) was added. The reaction mixture was heated under reflux for 3 hours then poured onto a beaker containing an ice/water mixture. The formed solid product was collected by filtration and dried obtaining pale yellow crystals (from ethanol).

Method B: To a solution of compound **14** (2.82 g, 0.01 mol) in 1,4-dioxane (40 mL) containing triethylamine (0.5 mL), elemental sulfur (0.32 g, 0.01 mol) was added. The reaction mixture was heated under reflux for 2 h then poured onto ice/water containing few drops of hydrochloric acid. The formed solid product was collected by filtration. 

Yield: 1.95g, 62% (method A) and 2.20 g, 70% (method B), m.p. 112 °C; IR (KBr) υ/cm^−1^: 3400-3300 (NH_2_, NH), 3068 (CH-aromatic), 2886 (CH_3_), 2250 (CN), 1690 (C=O), 1638 (C=C); MS *m/z* (%) 314 [M^+^, 2.19 %]; ^1^H-NMR δ2.29-2.31 (m, 8H, 4CH_2_), 2.49 (s, 3H, CH_3_), 4.25 (s, 2H, NH_2_), 7.41-8.88 (m, 4H, pyridine-H), 10.76 (s, 1H, NH);^13^C-NMR δ: 13.8 (CH_3_), 22.8, 23.6, 24.1, 24.9 (4 CH_2_), 116.5, 122.6, 124.8, 130.2, 150.6, 151.8 (thiophene, pyridine C), 163.6 (C=O), 170.0 (C=N); Anal. Calcd. for C_16_H_18_N_4_OS (314.41): C, 61.12; H, 5.77; N, 17.82%. Found: C, 60.91; H, 6.01; N, 17.85%.

*2-Amino-5,6-dihydro-N'-(1-(pyridin-3-yl)ethylidene)-4H-cyclopenta[b]thiophene-3-carbohydrazide* (**13**). To a solution of **3 **(2.02 g, 0.01 mol) in ethanol (40 mL) containing triethylamine (1.0 mL) and elemental sulfur (0.32 g, 0.01 mol), cyclopentanone **12** (0.98 g, 0.01 mol) was added. The reaction mixture was heated under reflux for 3 h then poured onto a beaker containing an ice/water mixture. The formed solid product was collected by filtration and dried obtaining pale yellow crystals (from ethanol).Yield: 1.82 g, 61%, m.p. 140-144 °C; IR (KBr) υ/cm^−1^: 3450-3300 (NH_2_, NH), 3080 (CH-aromatic), 2890 (CH_3_), 2250 (CN), 1690 (C=O); MS *m/z* (%) 300 [M^+^, 0.34 %]; ^1^H-NMR δ2.28-2.35 (m, 6H, 3CH_2_), 2.48 (s, 3H, CH_3_), 4.25 (s, 2H, NH_2_), 7.41-8.99 (m, 4H, pyridine-H),10.78 (s, 1H, NH);^ 13^C-NMR δ: 14.0 (CH_3_), 20.8, 24.6, 26.9 (3 CH_2_), 116.9, 123.0, 124.6, 133.1, 150.1, 151.4 (thiophene, pyridine C), 163.4 (C=O), 169.8 (C=N); Anal. Calcd. for C_15_H_16_N_4_OS (300.3): C, 59.98; H, 5.37; N, 18.65%. Found: C, 59.93; H, 5.39; N, 18.81%

*2-Cyano-2-cyclohexylidene-N'-(1-(pyridin-3-yl)ethylidene)acetohydrazide* (**14**). Equimolar amounts of compound **3** (2.02 g, 0.01 mol) and cyclohexanone **10 **(0.98 g, 0.01 mol) were heated in an oil bath at 140 °C for 1 h in presence of ammonium acetate. After cooling the reaction mixture, it was heated in ethanol, then poured into ice/water mixture and the formed solid product was collected by filtration and dried to give pale yellow crystals (from ethanol). Yield: 1.52 g, 54% , m.p. 145-146 °C; IR (KBr) υ/cm^−1^: 3355-3370 (NH), 3067 (CH-aromatic), 2930 (CH_3_), 2200 (CN), 1675 (C=O); MS *m/z* (%) 282 [M^+^, 0.40 %]; ^1^H-NMR δ 2.26-2.34 (m, 10H, 5CH_2_), 2.31(s, 3H, CH_3_), 7.41-8.99 (m, 4H, pyridine-H), 10.90 (s, 1H, NH);^ 13^C-NMR δ: 13.9 (CH_3_), 26.8, 27.4, 26.9, 27.0 (cyclohexane C), 93.0 (C=C), 116.8 (CN), 123.4, 126.8, 126.4, 150.5, 151.2 (pyridine C), 168.2 (C=N), 177.3 (C=O); Anal. Calcd. for C_16_H_18_N_4_O (282.34): C, 68.06; H, 6.43; N, 19.84%. Found: C, 67.85; H, 6.31; N, 20.22%

### 3.4. General Procedure for the Synthesis of *18* and *20*

Compound **3** (2.02 g, 0.01 mol) is dissolved in ethanol and a few sodium hydroxide pellets were added. Phenylisothiocyanate (**15**, 1.35, 0.01 mol) is then added and the solution is covered and left standing overnight. Equimolar amounts of either ethyl 2-bromoacetate (**17**) or ethyl 2-bromo-2-cyanoacetate (**19**) are stirred in the following day, and the solution is covered for another night, after which the reaction mixture is poured onto ice and the precipitated solid is filtered off.

*2-(4-Hydroxy-3-phenylthiazol-2(3H)-ylidene)-2-isocyano-N'-(1-(pyridin-3-yl)ethylidene) acetohydrazide* (**18**). Orange crystals (from ethanol). Yield: 2.43 g, 64%, m.p. 125-127 °C; IR (KBr) υ/cm^−1^: 3500-3370 (NH), 3400 (OH), 3100 (CH-aromatic), 2900 (CH_3_), 2189 (CN), 1675 (C=O); MS *m/z* (%) 377 [M^+^, 7.9 %]; ^1^H-NMR δ 2.22 (s, 3H, CH_3_), 7.46 (thiazole H-5),7.54-8.95 (C_6_H_5_-pyridine H), 8.80 (NH), 10.13 (OH); ^13^C-NMR δ: 14.3 (CH_3_), 93.0, 101.2 (C=C), 115.8 (CN), 119.2, 120.5, 121.8, 122.3, 124.0, 124.8, 127.6, 130.5, 133.2, 137.0, 150.7, 152.2 (C_6_H_5_, thiazole, pyridine C), 168.4 (C=N), 170.0 (C=O); Anal. Cald. for C_19_H_15_N_5_O_2_S (377.42): C, 60.46; H, 4.01; N, 18.56; S, 8.50. Found: C, 60.50; H, 4.01; N, 18.55; S, 8.48%.

*(2Z)-Ethyl-2-((1-(pyridin-3-yl)ethylideneaminocarbamoyl)(cyano)methylene)-4-cyano-3-phenylthiazolidine-4-carboxylate* (**20**). Orange crystals (from ethanol).Yield: 3.22 g, 70%, m.p. 125-127 °C; IR (KBr) υ/cm^−1^: 3577-3370 (NH), 3067 (CH-aromatic), 2930 (CH_3_), 2200 (CN), 1675 (C=O); MS *m/z* (%) 461.1 [M^+^, 25.2 %]; ^1^H-NMR δ 1.56 (t, 3H, *J* = 7.02 Hz, CH_3_), 2.26 (s, 3H, CH_3_), 4.25 (q, 2H, *J* = 7.02 Hz, CH_2_), 6.47 (s, 2H, thiazole, CH_2_), 7.08-8.12 (m, 9H, C_6_H_5_, pyridine-H), 10.72 (s, 1H, NH); ^13^C-NMR δ : 13.9, 14.5 (2 CH_3_), 40.2 (thiazole CH_2_), 58.9 (ester CH_2_), 93.3, 101.6 (C=C), 116.0, 116.7 (2 CN), 119.2, 120.3, 121.2, 121.8, 122.0, 124.7, 125.3, 133.5, 133.9, 150.4, 152.1 (C_6_H_5_, thiazole, pyridine C), 160.2, 164.5 (2 C=O), 168.9 (C=N); Anal. Calcd. for C_23_H_20_N_6_O_3_S (460.51): C, 59.99; H, 4.38; N, 18.25; S, 6.96%. Found: C, 60.11; H, 4.42; N, 18.13; S, 7.26%.

*4-Amino-2,3-dihydro-3-phenyl-N'-(1-(pyridin-3-yl)ethylidene)-2-thioxothiazole-5-carbohydrazide* (**21**). To a solution of **3 **(2.02 g, 0.01 mol) in ethanol (40 mL) containing triethylamine (1.0 mL) and elemental sulfur (0.32 g, 0.01 mol), phenylisothiocyanate (**15**, 1.35 g, 0.01 mol) was added. The reaction mixture was heated under reflux for 3 h then poured onto a beaker containing an ice/water mixture. The formed solid product was collected by filtration and dried obtaining yellow crystals (from ethanol). Yield: 2.46 g, 67% ,m.p. 164-167 °C; IR (KBr) υ/cm^−1 ^: 3465-3300 (NH_2_, NH), 3166 (CH-aromatic), 2980 (CH_3_), 1680 (C=O), 1658 (C=N), 1466 (C=C), 1241(C=S); MS *m/z* (%) 369.9 [M^+^, 13.27%]; ^1^H-NMR δ 2.33 (s, 3H, CH_3_), 3.31(s, 2H, NH_2_),7.28-7.38 (m, C_6_H_4_-pyridine H), 10.67 (s, 1H, NH);^ 13^C-NMR δ: 14.2 (CH_3_), 120.3, 122.5, 124.1, 127.9, 128.3, 130.1, 133.4, 138.9, 150.0, 152.3 (C_6_H_5_, thiazole, pyridine C), 168.2 (C=N), 170.2 (C=O), 180.3 (C=S); Anal. Calcd. for C_17_H_15_N_5_OS_2_ (369.46): C, 55.26; H, 4.09; N, 18.96; S, 17.36 %. Found: C, 55.40; H, 4.31; N, 19.15; S, 17.60%.

### 3.5. General Procedure for the Synthesis of *23a* or *23b*

To a solution of **3 **(2.02 g, 0.01 mol) in 1,4-dioxane (20 mL) either 2-benzylidenemalononitrile (**22a**, 1.54 g, 0.01 mol) or ethyl 2-cyano-3-phenylacrylate (**22b**, 2.01 g, 0.01 mol) was added. The reaction mixture was heated under reflux for 3 h then poured onto a beaker containing an ice/water mixture. The formed solid product was collected by filtration and dried.

*1-(1-Phenylethylideneamino)-6-amino-1,2-dihydro-2-hydroxy-4-phenylpyridine-3,5-dicarbonitrile* (**23a**). White crystals (from ethanol). Yield: 2.40 g, 68%, m.p. >300 °C; IR (KBr) υ/cm^−1^: 3458-3328 (NH_2_, NH), 3215 (CH-aromatic), 2890 (CH_3_), 2192, 2225 (2 CN), 1688 (C=O), 1640 (C=N); MS *m/z* (%) 355 [M^+^, 4.9 %]; ^1^H-NMR δ 2.301 (s, 3H, CH_3_), 3.56 (s, 2H, NH_2_), 7.46-9.22 (m, C_6_H_5_, pyridine H); ^13^C-NMR δ: 18.9 (CH_3_), 116.9, 118.0 (2 CN), 110.2, 118.9, 120.6, 128.4, 137.2, 150.2, 152.4 (two pyridine C), 168.9 (C=N), 172.3 (C=O); Anal. Calcd. for C_20_H_14_N_6_O (354.36): C, 67.79; H, 3.98; N, 23.72%. Found: C, 67.80; H, 4.00; N, 23.71%.

*Ethyl-1-(1-phenylethylideneamino)-6-amino-5-cyano-1,2-dihydro-2-hydroxy-4-phenylpyridine-3-carboxylate* (**23b**). White crystals (from ethanol).Yield: 1.96 g, 49%, m.p. 255-259 °C; IR (KBr) υ/cm^−1^: 3464-3339 (NH_2_), 3200 (CH-aromatic), 2890 (CH_3_), 2180 (CN), 1680 (C=O), 1640 (C=N); MS *m/z* (%) 401 [M^+^, 2.8 %]; ^1^H-NMR δ: 1.65 (t, 3H, *J* = 6.83 Hz, CH_3_), 2.31 (s, 3H, CH_3_), 3.56 (s, 2H, NH_2_), 4.18 (q, 2H, *J* = 6.83 Hz, CH_2_), 7.27-8.79 (m, C_6_H_5_, pyridine H); ^13^C-NMR δ: 13.7, 19.0 (2 CH_3_), 59.8 (CH_2_), 116.9 (CN), 108.0, 117.4, 120.3, 124.6, 125.1, 127.0, 132.0, 138.3, 150.2, 152.4 (C_6_H_5_, two pyridine C), 167.3 (C=N), 170.1, 172.6 (2C=O); Anal. Calcd. for C_22_H_19_N_5_O_3_ (401.42): C, 65.83; H, 4.77; N, 17.45%. Found: C, 66.04; H, 4.83; N, 17.61%.

## 4. Conclusions

In this work, cyanoacetylhydrazine (**1**) reacted with 3-acetylpyridine (**2**) to afford the hydrazide-hydrazone derivative **3**. The latter was reacted with different reagents to give coumarin, pyridine, thiazole and thiophene derivatives. The antitumor evaluations of the newly synthesized products were carried out, showing that both the hydrazide-hydrazone derivative **3** and the benzylidene derivative **5c** have the highest inhibitory effects.
